# An ecological investigation of average and peak external load intensities of basketball skills and game-based training drills

**DOI:** 10.5114/biolsport.2023.119291

**Published:** 2022-09-15

**Authors:** Pierpaolo Sansone, Lorenzo Gasperi, Bojan Makivic, Miguel Angel Gomez-Ruano, Antonio Tessitore, Daniele Conte

**Affiliations:** 1 Faculty of Sport Sciences, Catholic University of Murcia, Murcia, Spain; 2 UCAM Research Center for High Performance Sport, Catholic University of Murcia, Murcia, Spain; 3 Facultad de Ciencias de La Actividad Física y Del Deporte, Universidad Politécnica de Madrid, Madrid, Spain; 4 University of Applied Sciences Wiener Neustadt, Austria; 5 Department of Movement, Human and Health Sciences, University of Rome ‘Foro Italico’, Rome, Italy; 6 Institute of Sport Science and Innovations, Lithuanian Sports University, Kaunas, Lithuania

**Keywords:** Worst-case scenario, Small-sided games, Training load, Team sports, Constraints

## Abstract

This study quantified average and peak external intensities of various basketball training drills. Thirteen youth male basketball players (age: 15.2 ± 0.3 years) were monitored (BioHarness-3 devices) to obtain average and peak external load per minute (EL · min^−1^; peak EL · min^−1^) during team-based training sessions. Researchers coded the training sessions by analysing the drill type (skills, 1vs1, 2vs2, 3vs0, 3vs3, 4vs0, 4vs4, 5vs5, 5vs5-scrimmage), court area per player, player’s involvement in the drill (in percentage), playing positions (backcourt; frontcourt) and competition rotation status (starter; rotation; bench). Separate linear mixed models were run to assess the influence of training and individual constraints on average and peak EL · min^−1^. Drill type influenced average and peak EL · min^−1^ (p < 0.05), but with different directions of effects. EL · min^−1^ was higher in skills and 4vs0 drills, while higher peak EL · min^−1^ values were obtained in 5vs5 and 5vs5-scrimmage. Similarly, EL · min^−1^ was higher when involvement % increased (p = 0.001), while there was an opposite trend for peak EL · min^−1^ (lower with higher involvement %). Court area per player influenced peak (p = 0.025) but not average demands. No effects were found for playing position or competition rotation status (all p > 0.05), except for a moderately higher EL · min^−1^ in starters compared to bench players. The external load intensities of basketball training drills substantially vary depending on the load indicator chosen, the training content, and task and individual constraints. Practitioners should not interchangeably use average and peak external intensity indicators to design training but considering them as separate constructs could help to gain a better understanding of basketball training and competition demands.

## INTRODUCTION

Game-based conditioning drills are an essential training mode in basketball and a preferred method for coaches to effectively train players [1–3]. Within a game-based drill, players can be exposed to the same multiple stimuli occurring during basketball games, such as sport-specific movements and technical actions (skills), tactical scenarios requiring cognitive processes and decision making, as well as high physical and physiological demands. Previous studies have demonstrated how training interventions focusing on small-sided games and ball drills can concurrently lead to physiological adaptations and improvements in physical performance as well as developing specific technical-tactical skills in basketball players [4, 5].

The effectiveness and time-efficiency of this training mode justify the increased interest shown by basketball researchers in the last decade. The possibility of manipulating an abundance of training constraints, such as number of players [1, 2], court area [1, 2] training regimes [1–3, 6], time pressure [7] rules [8, 9] and tactics [7], offers a unique possibility for coaches to design variable training scenarios, closely related to the stochastic nature of basketball game play. However, this growing body of literature on game-based conditioning in basketball presents some limitations. Indeed, previous works encompassed research designs where players were alternatively exposed to different ball drills for a limited number of sessions, which possess a low ecological perspective, limiting the finding’s applicability in real-life training settings. By contrast, the analysis of performance during game-based conditioning drills and, more generally, team-based settings can vary [10, 11] due to the complex interactions among individual (e.g. physical capacities, fatigue), task (e.g. rules, court area, tactical content, playing positions) and environment (e.g. score line) constraints, which cannot be assessed in studies involving only a single or low number of game-based drills. Regarding individual constraints, for the same training drill different physical performances can be expected across players of different age. While most research has focused on professional or adult players, physical demands in youth basketball players have been investigated less, possibly due to limited financial interests and the consequent unavailability of monitoring technologies [12, 13]. Furthermore, a player’s involvement within a drill can vary significantly due to other variables not accounted for in the available literature, such as the number of players participating in the training session, interventions and breaks called by coaches, and other rules applied. Therefore, there is a need for a real-life context, ecological description of the demands of game-based conditioning drills during youth basketball training.

Another important aspect recently highlighted in basketball research is the analysis of peak demands, or most demanding scenarios [14, 16]. Studies across various team sports have suggested that quantifying external load intensities of training and competition based on classic average methods might underestimate the most demanding passages players have to engage in [14, 15]. These passages are defined as the most intense activity period (for an arbitrarily selected time frame, typically 1 minute) for a player within training or competition settings [14, 16, 17]. Examples of basketball scenarios characterized by peak external intensities could be multiple ball possessions with no basket or stoppages, or repeated basket-to-basket fast break actions. Importantly, these high-intensity moments have been suggested to be significantly influenced by contextual-related factors (e.g. technical-tactical aspects, score line, playing time, game quarter, game importance), that could be more frequently present during key competitions or game phases [14, 16, 17]. Therefore, these passages are of particular interest as they can be decisive to succeed in games. On the other hand, they might also indicate moments of very high physical loads imposed on the players’ bodies, with potential consequences for load management and injury risk. In this perspective, the most common approach to calculating the average external intensity (e.g. external load per minute, EL · min^−1^) for a whole game, quarter or training drill might include stoppages, breaks called by the coach in training, as well as phases of less intense activity [14]. In contrast, a peak demands approach would allow practitioners to detect with more precision the most demanding passages of training and competition. For instance, Fox et al. [14] reported 3 to 4 times higher EL · min^−1^ in basketball training and games when considering peak indicators, compared to average ones. Notably, Garcia et al. [16] found that basketball training fails to match the peak external intensities of competition, which are 6 to 35% higher. Therefore, accurately quantifying the demands imposed on basketball players appears essential for accurate training design and performance optimization. To the best of our knowledge, only a few studies have evaluated the most demanding scenarios in basketball and mostly in competition settings [14–17], with less information available on training [14, 16] or youth players [19], calling for further research. Additionally, there has been recent criticism of the worst-case scenario approach and its interpretation in team sport training [18] with limitations identified in the high variability of these peak intensity moments, which derives from the multiple physical, technical and contextual factors that come into play during sport performance. There appears to be a need for a better understanding of the average and peak external intensity indicators in team sports, and especially for youth players, about which less information is available despite the crucial importance of the formative years for players’ development. Therefore, the aim of this study was to evaluate the average and peak external load intensities of multiple basketball training drills in youth players, by using an ecological approach and considering different training (drill type, court area per player, player involvement percentage) and individual (playing position, competition rotation status) constraints.

## MATERIALS AND METHODS

### Participants

Fifteen youth male basketball players (backcourt [n = 6]: age: 15.1 ± 0.4 years; height: 173 ± 0.1 cm; body mass: 61.0 ± 6.3 kg; and training experience: 7.2 ± 2.5 years; frontcourt [n = 9]: age: 15.3 ± 0.2 years; height: 180 ± 0.5 cm; body mass: 69.8 ± 9.9 kg; and training experience: 7.8 ± 2.0 years) from a team participating in a highly ranked regional youth league organized by the Italian Basketball Federation (Federazione Italiana Pallacanestro, FIP) were recruited for this study. The team’s weekly schedule featured 3 teambased training sessions (70–90 min) and 1 official game. Players and their parents/guardians were informed about the monitoring procedures, and one of their parents/guardians signed a consent form before the start of the data collection. Players monitored in < 4 training sessions were excluded from the study [12]. In total, 13 players (n = 6 backcourts, n = 7 frontcourts) were finally included, with 391 individual data points of distinct training drills collected.

### Design

A descriptive design was adopted in this study to investigate the team’s training sessions during 10 in-season weeks (October-December). The training content of the sessions monitored (match-day-5, MD-5, and MD-2) was mainly focused on game-based conditioning and tactical drills, played on different court areas (full-court, half-court, modified), and individual skills training. Physical conditioning without the ball was sparsely implemented across the monitoring period, and therefore was not included in the study. Additionally, warm-up and cool-down procedures were also excluded from the analyses.

### Procedures

Players were monitored with BioHarness 3 microsensors (Zephyr Medtronic, Boulder, CO) to measure the average and peak external load intensity, by calculating the Impulse Load. This variable is calculated as the sum of areas under the 3-axis accelerometry curves (expressed in newtons per second (N · s)). Impulse Load has shown robust agreement with other commonly used indicators of external load obtained by microsensors (such as PlayerLoad) in team sports [20], as well as recently used in basketball research [12, 21].

Average external intensities of each training drill were calculated as Impulse Load per minute. To avoid confusion, this variable will be referred in this article as EL · min^−1^. Peak external intensity (peak EL · min^−1^) was calculated as the most demanding 1-minute passage during a given training drill [14, 16].

A summary of the variables considered in this study is presented in Table 1. The research group accurately coded the training sessions of the team by analysing: 1) the duration of each drill, in minutes; 2) the drill type; 3) the number of players on (e.g. actively playing) and off the court (e.g. waiting for their turn) to calculate the percentage of participation during the drill, which was defined as involvement percentage (%); 4) the court area on which the drill was played [22] (half-court, full-court, quarter-court and other measures used by the coaching staff) to calculate the court area per player (in m^2^). 5vs5-scrimmage refers to a 5vs5 played with competition-like rules and settings (court area, score count, fouls and free throws, shot clock). Additionally, playing position (backcourt; frontcourt) and competition rotation status were considered. Players were classified, according to the playing time registered by the coaching staff during official games, as starters (the five players starting the game and accumulating higher playing times), rotation (players who are on the bench at the start of the game but accumulate moderate playing times) or bench players (those with the lowest playing times). The data collection followed an ecological approach, with no interventions from the research team during the monitored training sessions.

**TABLE 1:** Description of the factors included in the linear mixed models

**Drill type**	Skills training (dribbling, shooting, passing, post-moves); 1vs1; 2vs2; 3vs0; 3vs3; 4vs0; 4vs4; 5vs5; 5vs5-scrimmage
**Court area per player**	in m^2^
**Involvement %**	% of involvement of players during the drill; e.g. 4vs4, 8 players in action, 4 players waiting outside the court = 66.7%
**Playing position**	Backcourt; frontcourt
**Competition rotation status**	Starter; rotation; bench

### Statistical analyses

Statistical analysis was performed using R version 4.0.3, RStudio version 1.4.1103, and the package *lmer* version 1.1–28. Two separate linear mixed models were used to evaluate the influence of drill type, court area per player, involvement %, playing position and competition rotation status on the average (EL · min^−1^) and peak (peak EL · min^−1^) physical demands encountered by basketball players (inserted in the model as a random effect) [12, 14]. Three variables are categorical, multi-level, having nine (drill type), three (competition rotation status) and two (playing position) factors, respectively. Therefore, a post-hoc Bonferroni correction was applied for p-values when performing all combinations of pairwise comparisons. Subsequently, a second linear mixed model was applied for each dependent variable to assess a possible interaction effect, by including only those variables which showed a significant main effect [23]. Data are presented as mean and standard error (SE).

Effect sizes (ES) were calculated using Cohen’s *d*, and interpreted as: < 0.20, trivial; 0.20 to 0.59, small; 0.60 to 1.19, moderate; 1.20 to 1.99, large; and > 2.0, very large [24].

## RESULTS

Significant effects on EL · min^−1^ were found for drill type (p < 0.05) and involvement % (p = 0.001) while no effects were found for playing positions, court area per player or competition rotation status (all p > 0.05). Regarding peak EL · min^−1^, there were significant effects of drill type and court area per player (all p < 0.05), while no differences were found for involvement %, playing positions or competition rotation statuses (all p > 0.05).

**FIG. 1:**
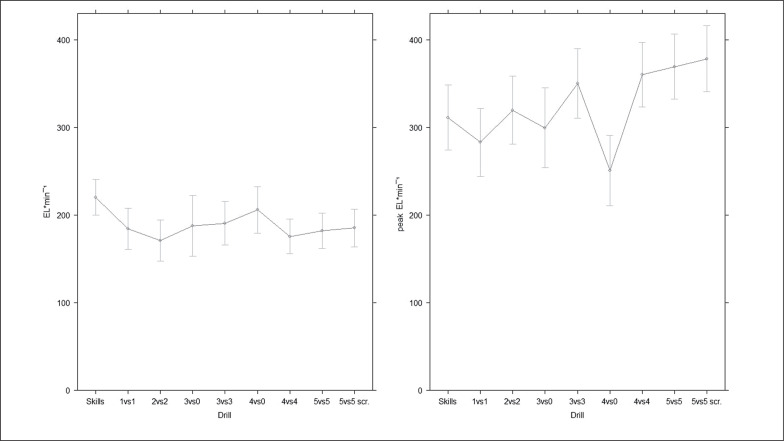
Average and peak external intensities across drill types.

**FIG. 2:**
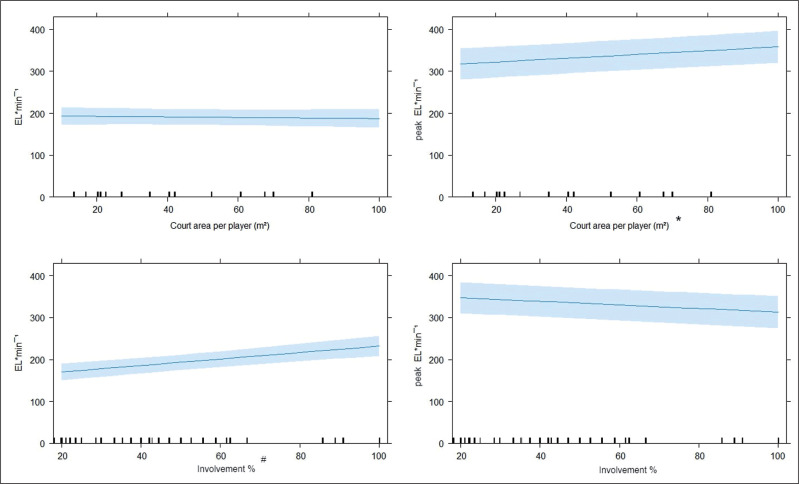
Influence of court area per player and involvement % on average and peak external intensities. * significant main effect of court area per player (p = 0.025); ^#^ significant main effect of involvement % (p = 0.001).

Figures 1 and 2 present average and peak external intensities across different ball drills, court areas per player and involvement %. The drills leading to higher EL · min^−1^ were skills (220 ± 20 N · s) and 4vs0 (206 ± 13 N · s); conversely, higher peak EL · min^−1^ was registered in 5vs5 (369 ± 19 N · s) and 5vs5-scrimmage (378 ± 19 N · s). Table 2 presents pairwise comparisons between drills with significantly different average or peak EL · min^−1^. Higher involvement % led to greater EL · min^−1^ (p = 0.001), while there was an opposite trend for peak EL · min^−1^ (lower with higher involvement %) (non-significant, p = 0.140). Larger court area per player elicited higher peak (p = 0.025) but not average external intensities. Regarding playing position, no significant differences were found for EL · min^−1^ (backcourts: 182 ± 23 N · s, frontcourt: 198 ± 24 N · s) (p = 1, ES: 0.38, small) and peak EL · min^−1^ (backcourt: 339 ± 39 N · s; frontcourt: 333 ± 38 N · s) (p = 1, ES: 0.06, trivial). Additionally, differences in EL · min^−1^ and peak EL · min^−1^ across competition rotation statuses were not statistically significant (all p > 0.05); however, there was a moderate (ES: 0.66) EL · min^−1^ difference between starters (203 ± 28 N · s) and bench players (177 ± 31 N · s), while small differences between starters and rotation players (187 ± 34 N · s) (ES: 0.50), and rotation vs bench (ES: 0.28) players were identified. The effects of competition rotation status on peak EL · min^−1^ were small and non-significant (starters: 320 ± 50 N · s; rotation: 337 ± 64 N · s; bench: 357 ± 53 N · s) (all p > 0.05, ESs: 0.27–0. 38, small).

**TABLE 2:** Pairwise comparisons between drill types with significant differences and effect size estimations.

EL · min^-1^	p	ES	
Skills > 1vs1	< 0.001	0.46	Small
Skills > 2vs2	< 0.001	0.60	Moderate
Skills > 3vs0	< 0.05	0.22	Small
Skills > 4vs4	< 0.001	0.68	Moderate
Skills > 5vs5	< 0.001	0.54	Small
Skills > 5vs5 scr.	< 0.001	0.44	Small

**Peak EL · min^-1^**	**p**	**ES**	

5vs5 scr. > skills	< 0.001	0.90	Moderate
5vs5 scr. > 1vs1	< 0.001	1.04	Moderate
5vs5 scr. > 2vs2	< 0.001	0.66	Moderate
5vs5 scr. > 3vs0	< 0.001	0.53	Small
5vs5 scr. > 4vs0	< 0.001	1.16	Moderate
5vs5 > skills	< 0.001	0.87	Moderate
5vs5 > 1vs1	< 0.001	1.00	Moderate
5vs5 > 2vs2	< 0.001	0.61	Moderate
5vs5 > 3vs0	< 0.001	0.48	Small
5vs5 > 4vs0	< 0.001	1.11	Moderate
4vs4 > skills	< 0.001	0.80	Moderate
4vs4 > 1vs1	< 0.001	0.98	Moderate
4vs4 > 2vs2	< 0.001	0.51	Small
4vs4 > 3vs0	< 0.001	0.43	Small
4vs4 > 4vs0	< 0.001	1.13	Moderate
3vs3 > skills	< 0.001	0.43	Small
3vs3 > 1vs1	< 0.001	0.62	Moderate
3vs3 > 4vs0	< 0.001	0.77	Moderate
2vs2 > 1vs1	< 0.05	0.38	Small
2vs > 4vs0	< 0.001	0.59	Small
1vs1 > skills	< 0.05	0.38	Small
Skills > 4vs0	< 0.001	0.65	Moderate

The second linear mixed models for interactions effects (EL · min^−1^: drill type*involvement %; peak EL · min^−1^: drill type*court area per player) showed no interaction (all p > 0.05), with all effect sizes between trivial and small.

## DISCUSSION

In team sports, when monitoring training demands in game-based and collective drills, it is important to evaluate them in ecological conditions, considering their potentially high variability deriving from the complex interaction of individual, task and environmental constraints [10, 11, 18]. Through an ecological approach (no experimental conditions or interventions performed) this study described the average and peak physical demands of skills and game-based conditioning drills in youth basketball training. The main findings were: 1) the same training constraints have different effects depending on the external load indicator considered (average or peak intensity); 2) the drills closer to competition contexts (4vs4, 5vs5) led to higher peak, but not average external intensities; 3) the involvement of players has opposite effects on average (higher with higher involvement) and peak (higher with lower involvement) external intensities; and 4) higher average but not peak physical performances were registered in players with higher competition playing times.

One of the main focuses of this study was to investigate how average and peak external intensity indicators are influenced by basketball training drills. Although several previous studies have described average peak and demands in basketball competition, [14–17] only two have monitored them during training drills [14, 16], with no detailed information on training content. Peak external intensities in this study were 84% (± 50%) higher than average ones. Interestingly, we found how, for the same training constraint, their effects on players’ external intensities can be substantially different. For example, average external load was higher in skills drills, while for the same drill the peak external intensity was among the lowest; importantly, average external intensity was higher with higher involvement % (higher work:rest ratio). Oppositely, players had higher peak physical performances when involvement % was lower (lower work:rest ratios, thus more rest). Altogether, our findings suggest that average and peak external intensities should be considered as two separate constructs, and therefore team sport practitioners should not choose one over the other.

During game-based and skills training, a variety of drills is typically implemented by coaches. For example, 1vs1 drills can help players improve their ability to generate an advantage (offensively) and place stress on individual defence, while 4vs4 and 5vs5 drills can be focused more on replicating competition-like tactical scenarios. In this study, nine different training drills were described. Regarding the average external intensity, 4vs0 and skills drills showed significantly higher average intensities. This fact can be possibly explained by the absence of opposition in these drills; therefore players might have determined their pace more freely and possibly adopted a more continuous and sustainable intensity. In fact, pacing strategies are an important determinant of team sport athletes’ performances during running-based training modes [25, 26], such as the ball drills monitored in this study. Differently, peak external intensities were registered in 4vs4 and 5vs5 drills, which resemble competition more (including in motivational aspects) and therefore stimulate players to reach higher peak performances. The 3vs3 drill, which is commonly implemented by basketball researchers and practitioners [3, 6, 7, 22], was also among the ones with higher peak EL · min^−1^. As our study involved youth players, it appears that this age group might be more stimulated by the ball and competition content of these drills, compared to those with no opposition, finally reaching higher peak intensities. On the other hand, adult professional players might approach the same training drills with a different mindset, leading to different average and peak external intensities. This is just a hypothesis, which we cannot confirm since basketball research on peak physical indicators is limited to competition studies. Our findings are in accordance with a recent study, which highlighted the considerable influence of tactical and contextual factors on peak external load indicators [18]. Figure 1 clearly shows how the influence of drill type on external intensity is quite divergent depending on whether average or peak indicators are taken into account. We therefore recommend practitioners to consider current findings when designing training drills for basketball players.

An important constraint coaches may manipulate in game-based conditioning drills is the court area on which the drill is performed. There is a general consensus that external loads are higher with larger court areas per player [2], and our findings partially confirm this. In fact, a significant main effect was found for peak external intensity, which was higher with increased m^2^ per player (see Figure 2). Thus, on larger court areas players tend to make greater physical efforts; however, the same was not found for average external intensity. As suggested above, it is possible that across the whole drill duration players pace themselves to maintain their exertion level [3, 25, 26], while in a 1-minute window, substantially higher peak intensities can be found when the court area is larger.

As we hypothesized, findings for average and peak external intensity were divergent when considering players’ involvement during training drills. Average external intensity was higher when players were more involved (higher involvement %) across the whole duration of the drill. Similarly, removing stoppages during 5v5 basketball drills has been shown to induce higher PlayerLoad per minute [27], possibly due to higher player involvement and less rest. When considering peak demands, the opposite appears to be true. Although the mixed model did not detect a statistically significant effect, a trend for higher peak external intensities with lower involvement % can be noticed (see Figure 2). Our findings support those of a recent basketball study [1], which showed how players reach higher peak external intensities when they accumulate lower playing times, both across the entire game and immediately before the peak intensity occurrence. More rest opportunities, or lower involvement %, such as in our study, can allow phosphocreatine resynthesis and reduce neuromuscular as well as perceptual fatigue [3, 26, 28], which can all help players to subsequently perform the multiple high-intensity actions [17] (sprints, decelerations, jumps) required for basketball performance. Therefore, the importance of manipulating work:rest ratios, controlling playing times and the use of substitutions to control training loads and to allow players to perform physically at their best during training and games is confirmed by our study.

We did not find any difference in either average or peak external intensity between backcourt and frontcourt players in this study. When comparing external loads of players of different positions, differences can be found when looking at specific load indicators, rather than global indicators such as Impulse Load (used in this study). For instance, backcourt players reach higher speeds [29] and cover greater distances [30], while frontcourt players perform more jumps and static exertions [31]. These differences are typically detected during game play, where players of different positions have to perform their specific tasks on court for a better collective performance. Differently, across multiple and varied training drills such as those monitored in this study, the influence of playing position and the players’ tasks on court might be mitigated by the training setting. This might be especially relevant and explain the current findings considering the sample involved (youth players), for which the training goal might be to be exposed to a greater variety of basketball scenarios [12] for better motor learning, with less emphasis on position-specific tasks.

Regarding competition rotation status, the higher the player’s importance [23], the greater was the average external intensity, especially when comparing starters and bench players (ES: moderate). However, there was no effect of competition rotation status on peak physical demands. Considering our findings and those of previous research [17], it is possible that average external load indicators are more influenced by players’ characteristics; for instance, starters might have better physical capacities and thus maintain higher physical intensities across the whole drill. By contrast, peak indicators are more variable [17, 18], and might be more influenced by other factors such as work:rest ratios, tactics and other contextual factors. Another possible explanations for the higher average external intensity of more important players (e.g. those who play more minutes) might be their more important roles in set plays during training drills. Specifically, these players can increase their activity on court and then their average external intensities, while at the same time they can reduce their rest on the court, and then limit their peak external performances.

Although this study provides useful insight for basketball practitioners, some limitations should be addressed. Firstly, only one external load measure (i.e. Impulse Load) was used in our study, limiting the generalization of our average and peak intensities to this measure. Furthermore, the comparison of our external intensity data is limited since this load indicator has not been extensively used in previous basketball studies. Secondly, no internal load responses (e.g. heart rate, sRPE) were adopted in our study, while their inclusion might be worthwhile since both average and peak external intensity phases have been suggested to elicit individualized internal responses in team sports [18] and youth athletes [32]. Therefore, future studies should focus on the analysis of average and peak intensity phases of other external load measures typically used in basketball training (e.g. accelerations, decelerations, jumps) and on the individual internal responses to both average and high-intensity phases.

## CONCLUSIONS

The physical demands of basketball training drills can substantially vary depending on the training load indicator chosen, the training content, and task and individual constraints. Specifically, an effect of drill type and rotation % was found for average external intensity measures, while an effect of drill type and court area per players was observed for peak external intensity. Conversely, no effect was found for playing position or competition rotation status for both external intensity measures. Overall, practitioners cannot use interchangeably average and peak external intensity measures to monitor basketball training sessions.

This study has several practical applications for basketball practitioners. Firstly, we found that the external load of average and peak intensity phases are two different measures of the training intensity during basketball training due to different trends for the dependent variables in this study. Based on the measure used to monitor the external intensity during training, different results of either average or peak indicators can be expected. Specifically, basketball coaches can manipulate the drill type based on the number of players involved and the involvement % to modify the average external intensity. Differently, modification of the number of players involved and court area per player should be used to modify the peak external load intensity. Implementing drills more similar to competition contexts (4vs4, 5vs5) can be used to increase players’ peak physical output intensity, while manipulation of work:rest ratios based on players’ involvement in the drills influences both average (higher with higher involvement) and peak (higher with lower involvement) external intensities. Concluding, we recommend that coaches manipulate training constraints to increase peak external intensity during skills training considering that technical movements during competitions are often performed during demanding game passages.

## References

[1] Conte D, Favero TG, Niederhausen M, et al. Effect of different number of players and training regimes on physiological and technical demands of ball-drills in basketball. J Sports Sci. 2016; 34(8):780–786. doi:10.1080/02640414.2015.1069384 26208533

[2] O’Grady CJ, Fox JL, Dalbo VJ, Scanlan AT. A systematic review of the external and internal workloads experienced during games-based drills in basketball players. Int J Sports Physiol Perform. 2020; 15(5):603–616. doi:10.1123/ijspp.2019-0785 32294618

[3] Sansone P, Tessitore A, Paulauskas H, et al. Physical and physiological demands and hormonal responses in basketball small-sided games with different tactical tasks and training regimes. J Sci Med Sport. 2019; 22(5):602–606. doi:10.1016/j.jsams.2018.11.017 30538078

[4] Delextrat A, Martinez A. Small-Sided Game Training Improves Aerobic Capacity and Technical Skills in Basketball Players. Int J Sports Med. 2014; 35(5):385–391. doi:10.1055/s-0033-1349107 24129991

[5] Maggioni MA, Bonato M, Stahn A, et al. Effects of ball drills and repeated-sprintability training in basketball players. Int J Sports Physiol Perform. 2019; 14(6):757–764. doi:10.1123/ijspp.2018-0433 30569788

[6] Sansone P, Tessitore A, Lukonaitiene I, et al. Technical-tactical profile, perceived exertion, mental demands and enjoyment of different tactical tasks and training regimes in basketball small-sided games. Biol Sport. 2020; 37(1):15–23. 32205906 10.5114/biolsport.2020.89937 PMC7075224

[7] Bredt SGT, Torres JO, Diniz LBF, et al. Physical and physiological demands of basketball small-sided games: The influence of defensive and time pressures. Biol Sport. 2020; 37(2):131–138. doi:10.5114/biolsport.2020.93038 32508380 PMC7249796

[8] Conte D, Favero TG, Niederhausen M, et al. Physiological and technical demands of no dribble game drill in young basketball players. J Strength Cond Res. 2015; 29(12):3375–3379. 26595130 10.1519/JSC.0000000000000997

[9] Ferioli D, Rucco D, Rampinini E, et al. Combined effect of number of players and dribbling on game-based-drill demands in basketball. Int J Sports Physiol Perform. 2020; 15(6):825–832. doi:10.1123/ijspp.2019-0645 32109883

[10] Clemente FM, Aquino R, Praça GM, et al. Variability of internal and external loads and technical/tactical outcomes during small-sided soccer games: A systematic review. Biol Sport. 2022; 39(3):647–672. doi:10.5114/biolsport.2022.107016 35959343 PMC9331334

[11] Poureghbali S, Arede J, Rehfeld K, Schöllhorn W, Leite N. Want to impact physical, technical, and tactical performance during basketball small-sided games in youth athletes? Try differential learning beforehand. Int J Environ Res Public Health. 2020; 17(24):1–12. doi:10.3390/ijerph17249279 PMC7763681 33322471

[12] Sansone P, Ceravolo A, Tessitore A. External, internal, perceived training loads and their relationships in youth basketball players across different positions. Int J Sports Physiol Perform. 2022; 17(2):249–255. doi: 10.1123/ijspp.2020-0962. 34583325

[13] Petway AJ, Freitas TT, Calleja-González J, Leal DM, Alcaraz PE. Training load and match-play demands in basketball based on competition level: a systematic review. PLoS One. 2020; 15(3): e0229212. doi:10.1371/journal.pone.0229212 32134965 PMC7058381

[14] Fox JL, Conte D, Stanton R, McLean B, Scanlan AT. The Application of Accelerometer-Derived Moving Averages to Quantify Peak Demands in Basketball. J Strength Cond Res. 2021; 35: s58–s63. doi:10.1519/jsc.0000000000003486 34846331

[15] Fox JL, Green J, Scanlan AT. Not All About the Effort ? A Comparison of Playing Intensities During Winning and Losing Game Quarters in Basketball. Int J Sport Physiol Perform. 2021; 16(9):1378–1381. 10.1123/ijspp.2020-0448 33662929

[16] García F, Schelling X, Castellano J, et al. Comparison of the most demanding scenarios during different in-season training sessions and official matches in professional basketball players. Biol Sport. 2022; 39(2):237–244. doi:10.5114/biolsport.2022.104064 35309543 PMC8919871

[17] Alonso Pérez-Chao E, Lorenzo A, Scanlan A, et al. Higher Playing Times Accumulated Across Entire Games and Prior to Intense Passages Reduce the Peak Demands Reached by Elite, Junior, Male Basketball Players. Am J Mens Health. 2021; 15(5):155798832110543. doi:10.1177/15579883211054353 PMC8558607 34720014

[18] Novak AR, Impellizzeri FM, Trivedi A, Coutts AJ, McCall A. Analysis of the worst-case scenarios in an elite football team: Towards a better understanding and application. J Sports Sci. 2021; 39(16):1850–1859. doi:10.1080/02640414.2021.1902138 33840362

[19] García F, Castellano J, Reche X, Vázquez-Guerrero J. Average Game Physical Demands and the Most Demanding Scenarios of Basketball Competition in Various Age Groups. J Hum Kinet. 2021 Jul 28; 79:165–174. doi: 10.2478/hukin-2021-0070. PMID: ; PMCID: . 34400996 PMC8336539

[20] Gómez-Carmona CD, Pino-Ortega J, Sánchez-Ureña B, Ibáñez SJ, Rojas-Valverde D. Accelerometry-based external load indicators in sport: Too many options, same practical outcome? Int J Environ Res Public Health. 2019; 16(24):1–13. doi:10.3390/ijerph16245101 PMC6950167 31847248

[21] Staunton C, Wundersitz D, Gordon B, Kingsley M. Discrepancies exist between exercise prescription and dose in elite women’s basketball pre-season. Sports. 2020; 8(5):1–10. doi:10.3390/sports8050070 PMC7281092 32438734

[22] Schelling X, Torres L. Accelerometer load profiles for basketball-specific drills in elite players. J Sport Sci Med. 2016; 15(4):585–591. PMC5131211 27928203

[23] Sansone P, Gasperi L, Tessitore A, Gomez MA. Training load, recovery and game performance in semi-professional male basketball: Influence of individual characteristics and contextual factors. Biol Sport. 2021; 38(2):207–217. doi:10.5114/biolsport.2020.98451 34079165 PMC8139347

[24] Hopkins WG, Marshall SW, Batterham AM, Hanin J. Progressive statistics for studies in sports medicine and exercise science. Med Sci Sports Exerc. 2009; 41(1):3–12. doi:10.1249/MSS.0b013e31818cb278 19092709

[25] Ferraz R, Gonçalves B, Tillaar R, et al. Effects of knowing the task duration on players ’ pacing patterns during soccer small-sided games. J Sports Sci. 2017; 00(00):1–7. doi:10.1080/24733938.2017.1283433 28134013

[26] Waldron M, Highton J. Fatigue and Pacing in High-Intensity Intermittent Team Sport: An Update. Sport Med. 2014; 44:1645–1658. doi:10.1007/s40279-014-0230-6 25047854

[27] Svilar L, Castellano J, Jukic I. Comparison of 5vs5 training games and match-play using microsensor technology in elite basketball. J Strength Cond Res. 2018; 33(7):1897–1903. 10.1519/JSC.0000000000002826 30204654

[28] Billaut F, Bishop D. Muscle fatigue in males and females during multiple-sprint exercise. Sport Med. 2009; 39(4):257–278. doi: 10.2165/00007256-200939040-00001 19317516

[29] Pino-Ortega J, Rojas-Valverde D, Gómez-Carmona CD, et al. Impact of Contextual Factors on External Load During a Congested-Fixture Tournament in Elite U ’ 18 Basketball Players. Front Psychol. 2019; 10(May):1–11. doi:10.3389/fpsyg.2019.01100 31156514 PMC6529817

[30] Scanlan A, Dascombe B, Reaburn P. A comparison of the activity demands of elite and sub-elite Australian men’s basketball competition. J Sports Sci. 2011; 29(11):1153–1160. doi:10.1080/02640414.2011.582509 21777151

[31] Delextrat A, Badiella A, Saavedra V, et al. Match activity demands of elite Spanish female basketball players by playing position. Int J Perform Anal Sport. 2017; 15(2):687–703. doi:10.1080/24748668.2015.11868824

[32] Lupo C, Capranica L, Cortis C, Guidotti F, Bianco A, Tessitore A. Session-RPE for quantifying load of different youth taekwondo training sessions. J Sports Med Phys Fitness. 2017 Mar; 57(3):189–194. doi: 10.23736/S0022-4707.16.06021-X. 26796074

